# Supplementary material for: What drives wolf preference towards wild ungulates? Insights from a multi-prey system in the Slovak Carpathians
Source: PLoS One. 2022 Jun 27;17(6):e0265386. doi: 10.1371/journal.pone.0265386 (PMC9236239; doi:10.1371/journal.pone.0265386)
Supplement: S1 Table — Estimated population sizes (number of individuals) of the main wild ungulate species within our study areas, Slovakia. (PDF) [file pone.0265386.s001.pdf]

S1 Table. Wild prey population estimations. Estimated population sizes (number of individuals) of the main wild ungulate species within our study areas, Slovakia.

Study areas<br>Species	Poľana PLA (ind.)	Vepor Mts (ind.)	Muráň NP (ind.)	Poloniny NP (ind.)	Total (ind.)
Red deer	1342	498	1271	201	3312
Roe Deer	1140	371	1073	275	2859
Wild boar	449	145	535	102	1231
Total (ind./area)	2931	1014	2879	578	7402
