# Supplementary material for: What drives wolf preference towards wild ungulates? Insights from a multi-prey system in the Slovak Carpathians
Source: PLoS One. 2022 Jun 27;17(6):e0265386. doi: 10.1371/journal.pone.0265386 (PMC9236239; doi:10.1371/journal.pone.0265386)
Supplement: S2 Table — Average population sizes (number of individuals) and densities (individuals/km2) of the main livestock species during 2015–2017 in all municipalities located within our study areas, Slovakia. (PDF) [file pone.0265386.s002.pdf]

**S2 Table. Livestock population size.** Average population sizes (number of individuals) and densities (individuals/km2) of the main livestock species during 2015 – 2017 in all municipalities located within our study areas, Slovakia.

| **Municipality** | **Area** (km²) | **Cattle** (ind.) | **Sheep** (ind.) | **Goats** (ind.) |
|---|---|---|---|---|
| Banská Bystrica [a] | 809 | 6425 | 17064 | 915 |
| Brezno [a] | 1265 | 7212 | 18965 | 1137 |
| Detva [a] | 449 | 4829 | 12680 | 539 |
| Poltár [a] | 476 | 4173 | 6546 | 531 |
| Revúca [a] | 730 | 3037 | 4134 | 421 |
| Rimavská Sobota [a] | 1471 | 12529 | 22245 | 1438 |
| Rožňava [b] | 1173 | 7149 | 8493 | 1401 |
| Snina [c] | 805 | 4226 | 2806 | 453 |
| Zvolen [a] | 759 | 7799 | 6150 | 802 |
| Total | 7937 | 57379 | 99083 | 7637 |
| Density (ind./km$^2$) | - | 7.2 | 12.5 | 1.0 |

County: [a] Banská Bystrica; [b] Košice; [c] Prešov
