# Supplementary material for: What drives wolf preference towards wild ungulates? Insights from a multi-prey system in the Slovak Carpathians
Source: PLoS One. 2022 Jun 27;17(6):e0265386. doi: 10.1371/journal.pone.0265386 (PMC9236239; doi:10.1371/journal.pone.0265386)
Supplement: S3 Table — Candidate logistic regression models explaining the probability that one species (red deer, wild boar, roe deer) would occur in winter wolf scat over other two species of wild ungulates. k–number of model components; LL–log-likelihood; AICc–Akaike Information Criterion for small sample sizes; w–AIC weight (best model highlighted in bold). (PDF) [file pone.0265386.s003.pdf]

S3 Table. Candidate logistic regression models. Candidate logistic regression models explaining the probability that one species (red deer, wild boar, roe deer) would occur in winter wolf scat over other two species of wild ungulates.

Species	Model	k	LL	ΔAICc	*w*
Red deer	log ($Density_{red\ deer}$) + Elevation + log (Deep snow %)	4	-181.5	**0.0**	**0.99**
	log ($Density_{red\ deer}$) + Elevation	3	-187.7	10.4	0.01
	log ($Density_{red\ deer}$)	2	-197.2	27.5	0.00
	Null	1	-201.6	34.3	0.00
Wild boar	log ($Density_{wild\ boar}$) + Elevation + log (Deep snow %)	4	-171.7	**0.0**	**0.99**
	log ($Density_{wild\ boar}$) + Elevation	3	-177.9	10.3	0.01
	log ($Density_{wild\ boar}$)	2	-187.2	25.4	0.00
	Null	1	-188.3	27.0	0.00
Roe deer	log ($Density_{red\ deer+wild\ boar}$) + Elevation + Study area	4	-120.2	**0.0**	**1.00**
	log ($Density_{red\ deer+wild\ boar}$) + Elevation	3	-131.5	13.5	0.00
	log ($Density_{red\ deer+wild\ boar}$)	2	-131.6	14.8	0.00
	log ($Density_{roe\ deer}$)	2	-131.8	16.3	0.00
	Null	1	-131.9	16.7	0.00

k – number of model components; LL – log-likelihood; AICc – Akaike Information Criterion for small sample sizes; w – AIC weight (best model highlighted in bold).
