# Supplementary material for: What drives wolf preference towards wild ungulates? Insights from a multi-prey system in the Slovak Carpathians
Source: PLoS One. 2022 Jun 27;17(6):e0265386. doi: 10.1371/journal.pone.0265386 (PMC9236239; doi:10.1371/journal.pone.0265386)
Supplement: S4 Table — Data set information used in the analysis: Sampling date (Month and Year) and prey item in each sample. (PDF) [file pone.0265386.s004.pdf]

S4 Table. Samples data set. Data set information used in the analysis: sampling date (Month and Year) and presence of prey in each sample.

Sample ID	Month	Year	Red deer	Roe deer	Wild boar	Sheep	Rodent	Bird	Brown hare	Total
1	Feb	2015	1	0	0	0	0	0	0	1
2	Feb	2015	1	0	0	0	0	0	0	1
3	Sep	2015	1	0	0	0	0	0	0	1
4	Sep	2015	1	0	0	0	0	0	0	1
5	Sep	2015	1	0	0	0	0	0	0	1
6	Sep	2015	0	0	1	0	0	0	0	1
7	Sep	2015	0	0	1	0	0	0	0	1
8	Sep	2015	0	0	1	0	0	0	0	1
9	Sep	2015	1	0	0	0	0	0	0	1
10	Sep	2015	1	0	0	0	0	0	0	1
11	Sep	2015	1	0	0	0	0	0	0	1
12	Sep	2015	1	0	0	0	0	0	0	1
13	Sep	2015	0	0	1	0	0	0	0	1
14	Sep	2015	0	0	1	0	0	0	0	1
15	Sep	2015	0	0	1	0	0	0	0	1
16	Sep	2015	1	0	0	0	0	0	0	1
17	Sep	2015	1	0	0	0	0	0	0	1
18	Oct	2015	1	0	0	0	0	0	0	1
19	Oct	2015	1	0	0	0	0	0	0	1
20	Oct	2015	0	0	1	0	0	0	0	1
21	Oct	2015	0	1	0	0	0	0	0	1
22	Oct	2015	0	1	0	0	0	0	0	1
23	Oct	2015	1	0	0	0	0	0	0	1
24	Oct	2015	1	0	0	0	0	0	0	1
25	Oct	2015	0	1	0	0	0	0	0	1
26	Feb	2016	1	0	0	0	0	0	0	1
27	Oct	2015	1	0	0	0	0	0	0	1
28	Dec	2015	1	0	0	0	0	0	0	1
29	Dec	2015	0	0	1	0	0	0	0	1
30	Dec	2015	0	1	0	0	0	0	0	1
31	Dec	2015	0	0	1	0	0	0	0	1
32	Dec	2015	0	0	1	0	0	0	0	1
33	Dec	2015	0	0	1	0	0	0	0	1
34	Dec	2015	1	0	0	0	0	0	0	1
35	Dec	2015	1	0	0	0	0	0	0	1
36	Dec	2015	0	0	1	0	0	0	0	1
37	Dec	2015	0	0	1	0	0	0	0	1
38	Dec	2015	0	0	1	0	0	0	0	1
39	Dec	2015	0	0	1	0	0	0	0	1
40	Dec	2015	0	0	1	0	0	0	0	1
41	Dec	2015	0	1	0	0	0	0	0	1
42	Dec	2015	0	0	1	0	0	0	0	1
43	Dec	2015	1	0	1	0	0	0	0	2
44	Dec	2015	0	0	1	0	0	0	0	1
45	Dec	2015	0	0	1	0	0	0	0	1
46	Dec	2015	0	0	1	0	0	0	0	1

47	Dec	2015	0	0	1	0	0	0	0	1
48	Dec	2015	0	1	0	0	0	0	0	1
49	Dec	2015	0	0	1	0	0	0	0	1
50	Dec	2015	0	0	1	0	0	0	0	1
51	Dec	2015	0	0	1	0	0	0	0	1
52	Dec	2015	0	0	1	0	0	0	0	1
53	Dec	2015	0	0	1	0	0	0	0	1
54	Dec	2015	0	0	1	0	0	0	0	1
55	Dec	2015	0	0	1	0	0	0	0	1
56	Dec	2015	1	0	1	0	0	0	0	2
57	Dec	2015	0	0	1	0	0	0	0	1
58	Dec	2015	0	0	1	0	0	0	0	1
59	Dec	2015	1	0	0	0	0	0	0	1
60	Dec	2015	1	0	0	0	0	0	0	1
61	Jan	2016	1	0	0	0	0	0	0	1
62	Feb	2016	0	1	0	0	1	0	0	2
63	Feb	2016	0	1	0	0	0	0	0	1
64	Feb	2016	0	0	0	0	1	0	0	1
65	Feb	2016	0	1	0	0	0	0	0	1
66	Feb	2016	1	0	0	0	0	0	0	1
67	Feb	2016	0	1	0	0	0	0	0	1
68	Feb	2016	0	1	0	0	0	0	0	1
69	Feb	2016	0	0	1	0	0	0	0	1
70	Feb	2016	0	1	0	0	0	0	0	1
71	Feb	2016	0	1	0	0	0	0	0	1
72	Feb	2016	1	0	0	0	0	0	0	1
73	Feb	2016	0	0	1	0	0	0	0	1
74	Feb	2016	0	1	0	0	0	0	0	1
75	Feb	2016	0	1	0	0	0	0	0	1
76	Feb	2016	1	0	0	0	0	0	0	1
77	Feb	2016	0	1	0	0	0	0	0	1
78	Feb	2016	0	1	0	0	0	0	0	1
79	Feb	2016	0	0	1	0	0	0	0	1
80	Feb	2016	1	0	0	0	0	0	0	1
81	Feb	2016	0	1	0	0	0	0	0	1
82	Feb	2016	1	0	0	0	0	0	0	1
83	Feb	2016	1	0	0	0	0	0	0	1
84	Feb	2016	0	0	0	1	0	0	0	1
85	Feb	2016	0	0	1	0	0	0	0	1
86	Feb	2016	1	0	1	0	0	0	0	2
87	Feb	2016	0	0	1	0	0	0	0	1
88	Feb	2016	1	0	0	0	0	0	0	1
89	Nov	2015	0	0	1	0	0	0	0	1
90	Dec	2015	1	0	0	0	0	0	0	1
91	Jan	2016	0	0	1	0	0	0	0	1
92	Jan	2016	0	0	1	0	0	0	0	1
93	Mar	2016	1	0	0	0	0	0	0	1
94	Mar	2016	1	0	0	0	0	0	0	1
95	Mar	2016	1	0	0	0	0	0	0	1
96	Mar	2016	0	1	0	0	0	0	0	1
97	Mar	2016	1	0	0	0	0	0	0	1

98	Mar	2016	0	0	1	0	0	0	0	1
99	Mar	2016	0	0	1	0	0	0	0	1
100	Mar	2016	0	0	1	0	0	0	0	1
101	Mar	2016	1	0	1	0	0	0	0	2
102	Mar	2016	1	0	0	0	0	0	0	1
103	Mar	2016	0	0	1	0	0	0	0	1
104	Mar	2016	0	0	1	0	0	0	0	1
105	Mar	2016	0	1	0	0	0	0	0	1
106	Mar	2016	1	0	0	0	0	0	0	1
107	Mar	2016	0	1	0	0	0	0	0	1
108	Mar	2016	1	0	0	0	0	0	0	1
109	Mar	2016	1	0	0	0	0	0	0	1
110	Mar	2016	0	0	1	0	0	0	0	1
111	Mar	2016	0	1	1	0	0	0	0	2
112	Mar	2016	1	0	0	0	0	0	0	1
113	Mar	2016	0	1	0	0	0	0	0	1
114	Mar	2016	0	0	1	0	0	0	0	1
115	Mar	2016	0	0	1	0	0	0	0	1
116	Mar	2016	1	0	0	0	0	0	0	1
117	Mar	2016	0	1	0	0	0	0	0	1
118	Mar	2016	0	1	0	0	0	0	0	1
119	Mar	2016	0	0	1	0	0	0	0	1
120	Mar	2016	1	0	0	0	0	0	0	1
121	Mar	2016	0	0	1	0	0	0	0	1
122	Mar	2016	1	0	0	0	0	0	0	1
123	Mar	2016	0	1	0	0	0	0	0	1
124	Mar	2016	1	0	0	0	0	0	0	1
125	Mar	2016	1	0	0	0	0	0	0	1
126	Mar	2016	1	0	0	0	0	0	0	1
127	Mar	2016	0	1	0	0	0	0	0	1
128	Mar	2016	0	0	1	0	0	0	0	1
129	Mar	2016	0	1	0	0	0	0	0	1
130	Mar	2016	0	0	1	0	0	0	0	1
131	Mar	2016	0	1	0	0	0	0	0	1
132	Mar	2016	0	0	1	0	0	0	0	1
133	Mar	2016	1	0	0	0	0	0	0	1
134	Mar	2016	1	0	0	0	0	0	0	1
135	Mar	2016	1	0	0	0	0	0	0	1
136	Mar	2016	1	0	0	0	0	0	0	1
137	Mar	2016	1	0	0	0	0	0	0	1
138	Mar	2016	0	0	0	0	0	1	0	1
139	Mar	2016	0	0	1	0	0	0	0	1
140	Mar	2016	1	0	0	0	0	0	0	1
141	Mar	2016	0	0	1	0	0	0	0	1
142	Mar	2016	1	0	0	0	0	0	0	1
143	Mar	2016	0	0	1	0	0	0	0	1
144	Mar	2016	0	1	0	0	0	0	0	1
145	Mar	2016	0	0	1	0	0	0	0	1
146	Mar	2016	0	1	0	0	0	0	0	1
147	Mar	2016	0	0	1	0	0	0	0	1
148	Mar	2016	0	0	1	0	0	0	0	1

149	Mar	2016	0	0	1	0	0	0	0	1
150	Mar	2016	1	0	0	0	0	0	0	1
151	Mar	2016	0	1	0	0	0	0	0	1
152	Mar	2016	1	0	0	0	0	0	0	1
153	Mar	2016	1	0	0	0	0	0	0	1
154	Mar	2016	0	1	0	0	0	0	0	1
155	Mar	2016	0	0	1	0	0	0	0	1
156	Mar	2016	0	0	1	0	0	0	0	1
157	Mar	2016	0	0	1	0	0	0	0	1
158	Mar	2016	0	0	1	0	0	0	0	1
159	Mar	2016	0	1	0	0	0	0	0	1
160	Mar	2016	0	0	1	0	0	0	0	1
161	Mar	2016	1	0	0	0	0	0	0	1
162	Mar	2016	1	0	0	0	0	0	0	1
163	Mar	2016	0	1	0	0	0	0	0	1
164	Mar	2016	1	0	0	0	0	0	0	1
165	Mar	2016	0	1	0	0	0	0	0	1
166	Mar	2016	0	0	1	0	0	0	0	1
167	Mar	2016	0	0	1	0	0	0	0	1
168	Mar	2016	1	0	0	0	0	0	0	1
169	Mar	2016	1	0	0	0	0	0	0	1
170	Mar	2016	0	1	0	0	0	0	0	1
171	Mar	2016	0	1	0	0	0	0	0	1
172	Mar	2016	1	0	0	0	0	0	0	1
173	Mar	2016	0	0	1	0	0	0	0	1
174	Feb	2016	0	0	1	0	0	0	0	1
175	Feb	2016	1	0	0	0	0	0	0	1
176	Feb	2016	1	0	0	0	0	0	0	1
177	Feb	2016	0	0	1	0	0	0	0	1
178	Feb	2016	0	1	0	0	0	0	0	1
179	Mar	2016	0	0	1	0	0	0	0	1
180	Mar	2016	1	0	0	0	0	0	0	1
181	Mar	2016	1	0	0	0	0	0	0	1
182	Mar	2016	0	0	1	0	0	0	0	1
183	Mar	2016	0	0	1	0	0	0	0	1
184	Mar	2016	0	0	1	0	0	0	0	1
185	Mar	2016	0	0	1	0	0	0	0	1
186	Mar	2016	0	0	1	0	0	0	0	1
187	Mar	2016	1	0	0	0	0	0	0	1
188	Mar	2016	1	0	0	0	0	0	0	1
189	Mar	2016	0	0	1	0	0	0	0	1
190	Mar	2016	0	0	1	0	0	0	0	1
191	Mar	2016	0	0	1	0	0	0	0	1
192	Mar	2016	1	0	0	0	0	0	0	1
193	Mar	2016	0	0	1	0	0	0	0	1
194	Mar	2016	0	0	1	0	0	0	0	1
195	Mar	2016	1	0	1	0	0	0	0	2
196	Mar	2016	1	0	0	0	0	0	0	1
197	Mar	2016	0	0	0	1	0	0	0	1
198	Mar	2016	0	0	1	0	0	0	0	1
199	Mar	2016	0	0	1	0	0	0	0	1

200	Mar	2016	0	1	0	0	0	0	0	1
201	Mar	2016	0	0	1	0	0	0	0	1
202	Mar	2016	1	0	0	0	0	0	0	1
203	Mar	2016	1	0	0	0	0	0	0	1
204	Mar	2016	1	0	0	0	0	0	0	1
205	Apr	2016	1	0	0	0	0	0	0	1
206	Apr	2016	0	1	1	0	0	0	0	2
207	Apr	2016	0	1	0	0	0	0	0	1
208	Apr	2016	1	0	0	0	0	0	0	1
209	Apr	2016	1	0	0	0	0	0	0	1
210	Feb	2017	1	0	0	0	0	0	0	1
211	Feb	2017	1	0	0	0	0	0	0	1
212	Feb	2017	0	1	0	0	0	0	0	1
213	Feb	2017	0	1	0	0	0	0	0	1
214	Jan	2017	1	0	0	0	0	0	0	1
215	Jan	2017	0	0	1	0	0	0	0	1
216	Jan	2017	0	1	1	0	0	0	0	2
217	Jan	2017	0	0	0	0	0	0	1	1
218	Feb	2017	0	0	1	0	0	0	0	1
219	Feb	2017	1	0	1	0	0	0	0	2
220	Feb	2017	1	0	0	0	0	0	0	1
221	Feb	2017	1	0	0	0	0	0	0	1
222	Feb	2017	0	0	1	0	0	0	0	1
223	Feb	2017	0	0	1	0	0	0	0	1
224	Feb	2017	0	0	1	0	0	0	0	1
225	Feb	2017	1	0	0	0	0	0	0	1
226	Feb	2017	1	0	0	0	0	0	0	1
227	Feb	2017	1	0	0	0	0	0	0	1
228	Feb	2017	0	1	0	0	0	0	0	1
229	Feb	2017	1	0	0	0	0	0	0	1
230	Feb	2017	0	0	1	0	0	0	0	1
231	Feb	2017	0	1	0	0	0	0	0	1
232	Feb	2017	1	0	0	0	0	0	0	1
233	Feb	2017	0	1	0	0	0	0	0	1
234	Feb	2017	1	0	0	0	0	0	0	1
235	Feb	2017	0	0	1	0	0	0	0	1
236	Feb	2017	1	0	0	0	0	0	0	1
237	Feb	2017	1	0	0	0	0	0	0	1
238	Feb	2017	1	0	0	0	0	0	0	1
239	Feb	2017	1	0	0	0	0	0	0	1
240	Feb	2017	1	0	0	0	0	0	0	1
241	Feb	2017	0	0	1	0	0	0	0	1
242	Feb	2017	0	0	1	0	0	0	0	1
243	Feb	2017	1	0	0	0	0	0	0	1
244	Feb	2017	0	1	0	0	0	0	0	1
245	Feb	2017	1	0	0	0	0	0	0	1
246	Feb	2017	1	0	0	0	0	0	0	1
247	Feb	2017	0	0	1	0	0	0	0	1
248	Feb	2017	0	0	1	0	0	0	0	1
249	Feb	2017	0	0	1	0	0	0	0	1
250	Feb	2017	1	0	1	0	0	0	0	2

251	Feb	2017	1	0	0	0	0	0	0	1
252	Feb	2017	1	0	0	0	0	0	0	1
253	Feb	2017	0	1	0	0	0	0	0	1
254	Feb	2017	0	0	1	0	0	0	0	1
255	Feb	2017	1	0	0	0	0	0	0	1
256	Feb	2017	1	0	0	0	0	0	0	1
257	Feb	2017	0	0	1	0	0	0	0	1
258	Feb	2017	0	1	0	0	0	0	0	1
259	Feb	2017	1	0	0	0	0	0	0	1
260	Feb	2017	1	0	0	0	0	0	0	1
261	Feb	2017	0	0	1	0	0	0	0	1
262	Feb	2017	1	0	1	0	0	0	0	2
263	Dec	2016	1	0	1	0	0	0	0	2
264	Dec	2016	0	0	1	0	0	0	0	1
265	Dec	2016	0	0	1	0	0	0	0	1
266	Dec	2016	1	0	0	0	0	0	0	1
267	Dec	2016	1	0	1	0	0	0	0	2
268	Dec	2016	1	0	0	0	0	0	0	1
269	Dec	2016	1	0	0	0	0	0	0	1
270	Jan	2015	1	0	1	0	0	0	0	2
271	Feb	2015	1	0	0	0	0	0	0	1
272	Feb	2015	1	0	0	0	0	0	0	1
273	Feb	2015	0	1	0	0	0	0	0	1
274	Apr	2015	1	0	1	0	0	0	0	2
275	Nov	2015	0	0	1	0	0	0	0	1
276	Dec	2015	1	0	0	0	0	0	0	1
277	Dec	2015	1	0	0	0	0	0	0	1
278	Jan	2017	1	0	0	0	0	0	0	1
279	Jan	2017	1	0	0	0	0	0	0	1
280	Feb	2017	1	0	0	0	0	0	0	1
281	Feb	2017	1	0	0	0	0	0	0	1
282	Feb	2017	1	0	0	0	0	0	0	1
283	Feb	2017	1	0	0	0	0	0	0	1
284	Feb	2017	1	0	0	0	0	0	0	1
285	Feb	2017	1	0	0	0	0	0	0	1
286	Feb	2017	1	0	0	0	0	0	0	1
287	Feb	2017	1	0	0	0	0	0	0	1
288	Feb	2017	1	0	0	0	0	0	0	1
289	Feb	2017	1	0	0	0	0	0	0	1
290	Feb	2017	1	0	0	0	0	0	0	1
291	Feb	2017	1	0	0	0	0	0	0	1
292	Feb	2017	1	0	0	0	0	0	0	1
293	Feb	2017	1	0	0	0	0	0	0	1
294	Feb	2017	1	0	0	0	0	0	0	1
295	Feb	2017	1	0	0	0	0	0	0	1
296	Feb	2017	1	0	0	0	0	0	0	1
297	Feb	2017	1	0	0	0	0	0	0	1
298	Feb	2017	1	0	0	0	0	0	0	1
299	Feb	2017	1	0	0	0	0	0	0	1
300	Feb	2017	1	0	0	0	0	0	0	1
301	Feb	2017	1	0	0	0	0	0	0	1

302	Feb	2017	1	0	0	0	0	0	0	1
303	Feb	2017	1	0	0	0	0	0	0	1
304	Feb	2017	1	0	0	0	0	0	0	1
305	Feb	2017	1	0	0	0	0	0	0	1
306	Feb	2017	1	0	1	0	0	0	0	2
307	Feb	2017	1	0	0	0	0	0	0	1
308	Feb	2017	1	0	0	0	0	0	0	1
309	Feb	2017	1	0	0	0	0	0	0	1
310	Feb	2017	1	0	0	0	0	0	0	1
311	Feb	2017	1	0	0	0	0	0	0	1
312	Feb	2017	1	0	0	0	0	0	0	1
313	Jan	2015	1	0	0	0	0	0	0	1
314	Feb	2015	1	1	0	0	0	0	0	2
315	Feb	2015	0	0	1	0	0	0	0	1
316	Apr	2015	1	0	0	0	0	0	0	1
317	Apr	2015	1	0	0	0	0	0	0	1
318	Apr	2015	1	0	0	0	0	0	0	1
319	Jan	2016	1	1	0	0	0	0	0	2
320	Feb	2016	1	0	0	0	0	0	0	1
321	Feb	2016	0	0	1	0	0	0	0	1
